# CtBP2 overexpression promotes tumor cell proliferation and invasion in gastric cancer and is associated with poor prognosis

**DOI:** 10.18632/oncotarget.15661

**Published:** 2017-02-24

**Authors:** Faxiang Dai, Yi Xuan, Jie-Jie Jin, Shengjia Yu, Zi-Wen Long, Hong Cai, Xiao-Wen Liu, Ye Zhou, Ya-Nong Wang, Zhong Chen, Hua Huang

**Affiliations:** ^1^ Department of Gastric Cancer and Soft Tissue Sarcoma, Fudan University Shanghai Cancer Center, Shanghai 200032, China; ^2^ Department of Oncology, Shanghai Medical College, Fudan University, Shanghai 200032, China; ^3^ Department of Hepatobiliary Surgery, Affiliated Hospital of Nantong University, Jiangsu Province, Nantong 226001, China

**Keywords:** CtBP2, gastric cancer, prognosis

## Abstract

C-terminal binding protein-2 (CtBP2), a transcriptional corepressor, has been reported to correlate with tumorigenesis and progression and predict a poor prognosis in several human cancers. However, few studies on CtBP2 in gastric cancer (GC) have been performed. In this research, we evaluated the correlations between CtBP2 expression and the clinicopathological characteristics, as well as prognosis of GC patients. The effects of silencing CtBP2 expression on GC cells biology activity were also assessed. The results showed that CtBP2 was overexpressed in GC tissues and closely correlated with poor differentiation, advanced tumor stage and poor prognosis in GC patients. CtBP2 induced epithelial-to-mesenchymal transition (EMT) and repressed PTEN to increase proliferation rate, migration, and invasion in GC cells. Silencing CtBP2 inhibited GC growth in nude mice model. In conclusion, CtBP2 is overexpressed in GC and may accelerate GC tumorigenesis and metastasis, which could represent an independent prognostic marker and promising therapeutic target for GC.

## INTRODUCTION

Gastric cancer (GC) is the fourth most common type of cancer with a low survival rate and represents an enormous burden on society worldwide [1]. At present, surgery combined with adjuvant or neoadjuvant chemotherapy is the major treatment for GC [2]. Despite improvements in treatment and a deeper understanding of GC, the 5-year relative survival rate of GC patients remains less than 30% [3]. Over the past ten years, explorations into the molecular mechanisms of carcinogenesis at the genomic level and targeted therapies have achieved significant progress [2, 4]. For example, the importance of HER2 (also known as EGFER, epidermal growth factor receptor) and the clinical use of trastuzumab (a monoclonal antibody against HER2) to treat GC patients are now widely accepted [5, 6, 7]. Furthermore, a number of other target antigens that could play important roles in GC tumorigenesis and progression have been under investigation [4, 8–11]. There is an urgent need to identify novel molecular antigens regulating GC progression that could serve as potential targets for GC therapy.

C-terminal binding protein-2 (CtBP2) is a known transcriptional corepressor and modulator of several essential cellular processes, and it has also been shown to activate tumorigenesis and tumor progression. CtBP2 induces the epithelial-to-mesenchymal transition (EMT), helps to repress a number of tumor suppressors (e.g., E-cadherin, PTEN, Ink4 family tumor suppressors), and functions as an antagonist of apoptosis [12, 13]. CtBP2 can bind to β-Catenin and participates in the regulation of Wnt signalling [14, 15]. CtBP2 is overexpressed in prostate cancer, hepatocellular carcinoma, and ovarian cancer with important effects on the biological activity and prognosis [16–18]. However, to date, no study has specifically analysed CtBP2 expression in GC or determined how it affects the biological characteristics of GC.

In this study, for the first time, we performed a comprehensive assessment of CtBP2 expression in GC and determined its association with specific GC clinicopathological characteristics. We found that CtBP2 expression was closely correlated with malignant behaviours and poor survival rate in GC patients. The biological effects of CtBP2 on cell proliferation, migration, and invasion in GC were also analysed. The results were in accordance with former studies [13–18]. CtBP2 might contribute to GC growth, metastasis and poor prognosis by promoting EMT and repressing PTEN. In conclusion, CtBP2 is a novel and effective predictive biomarker and potential therapeutic target for GC patients.

## RESULTS

### CtBP2 was overexpressed in GC tissues and correlated with poorly prognostic characteristics

**Figure 1:**
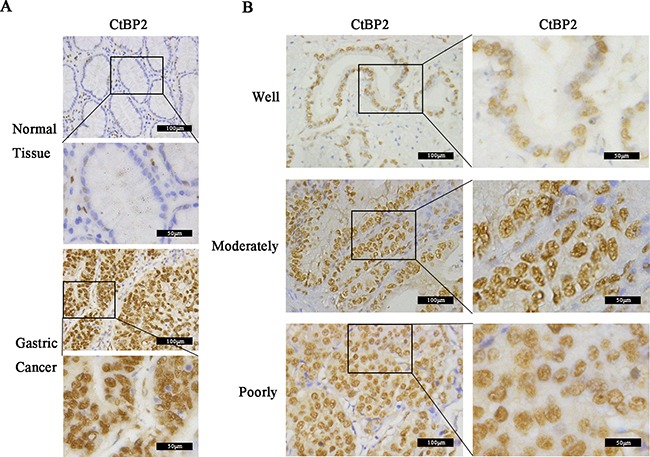
Immunohistochemical (IHC) analysis of CtBP2 expression in 352 gastric cancer (GC) and matched normal tissues Representative examples of IHC results. **A**. CtBP2 showed low expression in normal gastric tissues but high expression in GC tissues (magnification, ×200 and 400). **B**. CtBP2 expression in well-, moderately, and poorly differentiated GC tissues; the staining results were weak, moderate and strong, respectively (magnification, ×200 and 400).

**Figure 2:**
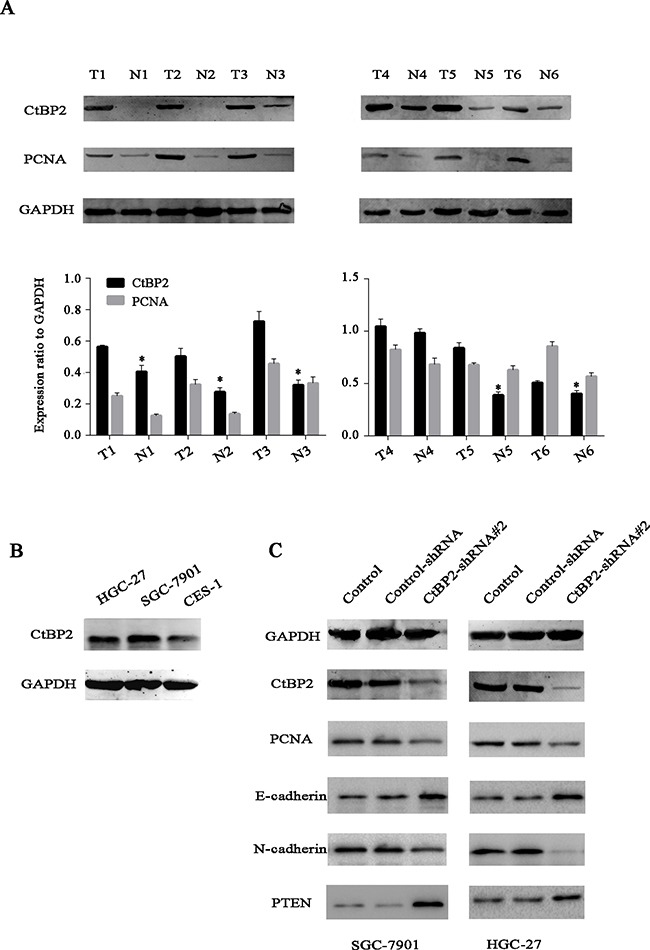
**A**. Western blotting analysis showed that the protein levels of CtBP2 were higher in six representative GC tissues than in matched adjacent normal gastric tissues. **B**. The expression of CtBP2 was higher in the GC cell lines (HGC-27 and SGC-7901) than in the normal gastric cell line GES1. **C**. GC cell lines (HGC-27 and SGC-7901) were transiently transfected with CtBP2-shRNA#2 for 48 h. Western blotting analysis demonstrated the silencing of CtBP2. Representative WB images showed the expression of PCNA, E-cadherin, N-cadherin, PTEN in untreated, Control-shRNA and CtBP2-shRNA#2 treated cells, respectively.

We examined CtBP2 expression in GC tissues and adjacent gastric tissues from 352 GC patients by IHC analysis. Results showed that CtBP2 was overexpressed in GC tissues and predominantly located in the nucleus. At the same time, CtBP2 showed markedly higher expression in poorly differentiated GC tissues than in well-differentiated ones. Representative IHC staining images of the GC samples were shown in Figure 1. We performed WB analysis of CtBP2 expression in 6 pairs of fresh GC tissues and adjacent normal gastric tissues The WB results showed that expression of CtBP2 was higher in GC tissues than in normal gastric tissues (Figure 2A). The correlations between high CtBP2 expression and the clinicopathological characteristics in the 352 cases of GC are shown in Table 1. High expression of CtBP2 in GC was significantly associated with differentiation (p = 0.035), TNM stage (p = 0.004), T classification (p < 0.001), lymph node metastasis (p = 0.002), distant metastasis (p = 0.002), vascular invasion (p = 0.004), and lymphatic invasion (p = 0.004). By contrast, no statistically significant relationships were found for age, gender, tumor size, Borrmann type, or carcinoembryonic antigen (CEA).

**Table 1:** Correlation between the expression of CtBP2 and clinicopathological characteristics of GC

Clinicopathological Characteristics	Total n	CtBP2 expression		p value
		Low n(%)	High n(%)	
**Total**	352	196(56)	156(44)	
**Age (years)**				
≦60	155	84(24)	71(20)	0.618
>60	197	112(32)	85(24)	
**Gender**				
Male	275	159(45)	116(33)	0.127
Female	77	37(11)	40(11)	
**Differentiation**				
Well	17	14(4)	3(1)	0.035*
Moderate	148	86(24)	62(18)	
Poor	187	96(27)	91(26)	
**Tumor size (cm)**				
<5	145	76(26)	69(20)	0.302
≧5	207	120(34)	87(25)	
**Borrmann Type**				
I	20	13(4)	7(2)	0.428
II	17	12(3)	5(1)	
III	302	163(46)	139(39)	
IV	13	8(2)	5(1)	
**TNM stage**^¶^				
I	11	9(3)	2(1)	0.004*
II	72	51(14)	21(6)	
III	221	109(31)	112(32)	
IV	48	27(8)	21(6)	
**T classification**				
T1	11	11(3)	0(0)	<0.001*
T2	15	13(4)	2(1)	
T3	27	20(6)	7(2)	
T4	299	152(43)	147(42)	
**Lymph node Metastasis**				
No	79	53(15)	26(7)	0.02*
Yes	273	143(41)	130(37)	
**Distant Metastasis**				
M0	257	130(37)	127(36)	0.02*
M1	95	66(19)	29(8)	
**Vascular invasion**				
No	166	106(30)	60(17)	0.004*
Yes	186	90(26)	96(27)	
**Lymphatic invasion**				
No	228	145(41)	83(24)	<0.001*
Yes	124	51(14)	73(21)	
**CEA**^†^				
Normal	253	144(41)	109(31)	0.456
Elevated	99	52(15)	47(13)	
**Survival status**				
Alive	233	157(45)	76(22)	<0.001*
Dead	119	39(11)	80(23)	

Abbreviations: Statistacal analyses were performed by the Pearson χ2 test, *p<0.05 was considered significant; ¶TNM pathological tumor-node-metastasis; †CEA carcinoembryonic antigen.

### Overexpression of CtBP2 predicts poor prognosis

Based on the TMA and IHC analyses of CtBP2 expression, the relationships between survival status and the clinicopathological characteristics were first assessed using χ^2^ tests (Table 2). The characteristics that had significant associations with survival status were tumor size (p = 0.001), TNM stage (p < 0.001), T classification (p = 0.02), lymph node metastasis (p < 0.001), distant metastasis (p < 0.001), vascular invasion (p < 0.001), lymphatic invasion (p < 0.001), and CtBP2 expression (p < 0.001).

**Table 2:** Survival status and clinicopathological characteristics in 352 himan GC specimens

Clinicopathological Characteristics	Total n	Survival status		p value
		Alive n(%)	Dead n(%)	
**Total**	352	233(66)	119(34)	
**Age (years)**				
≦60	155	101(29)	54(15)	0.717
>60	197	132(38)	65(18)	
**Gender**				
Male	275	178(51)	97(28)	0.272
Female	77	55(16)	22(6)	
**Differentiation**				
Well	17	13(4)	4(1)	0.264
Moderate	148	103(29)	45(13)	
Poor	187	117(33)	70(20)	
**Tumor size (cm)**				
<5	145	111(32)	34(10)	0.001*
≧5	207	122(35)	85(24)	
**Borrmann Type**				
I	20	13(4)	7(2)	0.266
II	17	15(4)	2(1)	
III	302	197(56)	105(30)	
IV	13	8(2)	5(1)	
**TNM stage**^¶^				
I	11	9(3)	2(1)	<0.001*
II	72	62(18)	10(3)	
III	221	137(39)	84(24)	
IV	48	25(7)	23(7)	
**T classification**				
T1	11	9(3)	2(1)	0.02*
T2	15	13(4)	2(1)	
T3	27	23(7)	4(1)	
T4	299	188(53)	111(32)	
**Lymph node Metastasis**				
No	79	67(19)	12(3)	<0.001*
Yes	273	166(47)	107(30)	
**Distant Metastasis**				
M0	257	208(59)	49(14)	<0.001*
M1	95	25(7)	70(20)	
**Vascular invasion**				
No	166	127(36)	39(11)	<0.001*
Yes	186	106(30)	80(23)	
**Lymphatic invasion**				
No	228	174(49)	54(15)	<0.001*
Yes	124	59(17)	65(18)	
**CEA**†				
Normal	253	175(50)	78(22)	0.059
Elevated	99	58(16)	41(12)	
**CtBP2 expression**				
Low	196	157(45)	39(11)	<0.001*
High	156	76(22)	80(23)	

Abbreviations: Statistacal analyses were performed by the Pearson χ2 test, *p<0.05 was considered significant; ¶TNM pathological tumor-node-metastasis; †CEA carcinoembryonic antigen.

**Figure 3:**
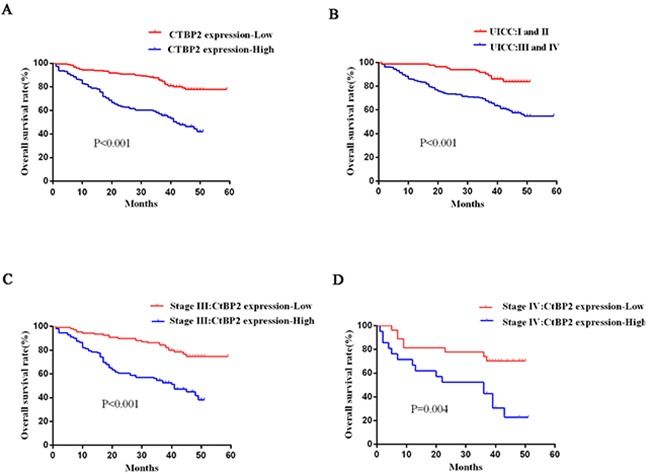
Analysis of GC patient survival prognosis using the Kaplan-Meier method **A**. The CtBP2 high-expression group (blue line) had significantly worse prognoses than the CtBP2 low-expression group (red line) (p < 0.001). **B**. The overall survival of stage I and II GC patients was significantly higher than for stage III and IV GC patients (p < 0.001). **C**, **D**. The CtBP2 high-expression group (blue line) had a significantly worse prognosis than the CtBP2 low-expression group (red line) at stage III or IV (p < 0.001 and p = 0.004, respectively).

Furthermore, Kaplan–Meier analyses were carried out to assess the associations between GC patient clinicopathological characteristics and survival prognosis. The log-rank test indicated that CtBP2 expression (p < 0.001), tumor size (p = 0.028), TNM stage (p < 0.001), vascular invasion (p < 0.001), and lymphatic invasion (p < 0.001) were meaningful prognostic indicators for overall survival (Table 3). Subsequent multivariate analysis confirmed that CtBP2 (p < 0.001), lymphatic invasion (p < 0.001), tumor size (p = 0.022), and TNM stage (p = 0.018) were independent prognostic indicators for GC patients (Table 3). CtBP2 high-expression group had significantly worse prognoses than the CtBP2 low-expression group which was learned from Kaplan–Meier survival curves (Figure 3).

**Table 3:** Univariate and multivariate analyses prognostic factors for overall survival in GC

Clinicopathological variables	Univariateanalysis	Multivariate analysis		
	p value	p value	HR	95%CI
Age (years): >60 vs ≦60	0.785	/	/	/
Gender: female vs male	0.411	/	/	/
Differentiation: well vs moderate, poor	0.085	/	/	/
Tumor size (cm): ≧5 vs <5	0.028*	0.022*	1.559	1.066-2.279
Borrmann type: I vs II vs III vs IV	0.315	/		
TNM stage: IV, III vs II, I	<0.001*	0.018*	2.098	1.133-3.882
T classification: T1 vs T2 vs T3 vs T4	0.033*	/	/	/
Lymph node Metastasis: Yes vs No	<0.001*	/	/	/
Distant Metastasis: Yes vs No	0.012*	/	/	/
Vascular invasion: Yes vs No	<0.001*	0.133	1.356	0.911-2.017
Lymphatic invasion: Yes vs No	<0.001*	<0.001*	2.144	1.473-3.120
CEA: Elevated vs Normal	0.516	/	/	/
CtBP2 expression: High vs Low	<0.001*	<0.001*	2.643	1.780-3.924

Abbreviation:*p<0.05 was considered significant. TNM stage contains T classification, lymph node metastasis and distant metastasis, they were not included in the multivariate analysis.

### CtBP2 might contribute to EMT and repressing PTEN in GC cells

We performed WB analysis of CtBP2 expression in GC cells, CtBP2 expression was also higher in both GC cell lines (HGC-27 and SGC-7901) than in the normal gastric cell line (GES1), as expected (Figure 2B). GC cell lines were transfected with different CtBP2-shRNA constructs; the CtBP2-shRNA#2 induced the most efficient knockdown, whereas the control-shRNA had no significant effect on CtBP2 expression (Figure 2C). Therefore, CtBP2-shRNA#2 and the control-shRNA were selected in subsequent experiments. We examined the expression of markers associated with tumor growth, EMT, tumor suppressor by WB. CtBP2 depletion downregulated PCNA and N-cadherin, while upregulated E-cadherin and PTEN in both cell lines.

### CtBP2 increased GC cell proliferation and migration, and invasion *in vitro*

**Figure 4:**
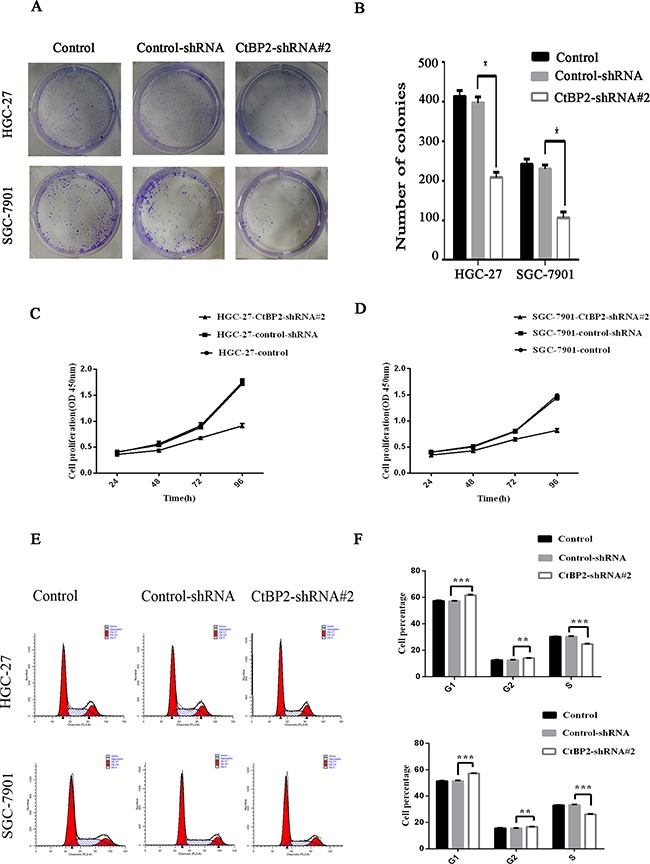
Knockdown of CtBP2 suppresses cell proliferation *in vitro* **A, B**. Colony formation assays showed that the downregulation of CtBP2 reduced the mean colony number in both HGC-27 and SGC-7901 cells. **C, D**. The downregulation of CtBP2 suppressed the growth rate of HGC-27 and SGC-7901 cells in CCK-8 cell proliferation assays. **E, F**. Cell cycle analysis of the role of CtBP2. Knockdown of CtBP2 resulted in an increase in the G1/S ratio in both HGC-27 and SGC-7901 cells (* p < 0.05, **p < 0.01, *** p < 0.001).

**Figure 5:**
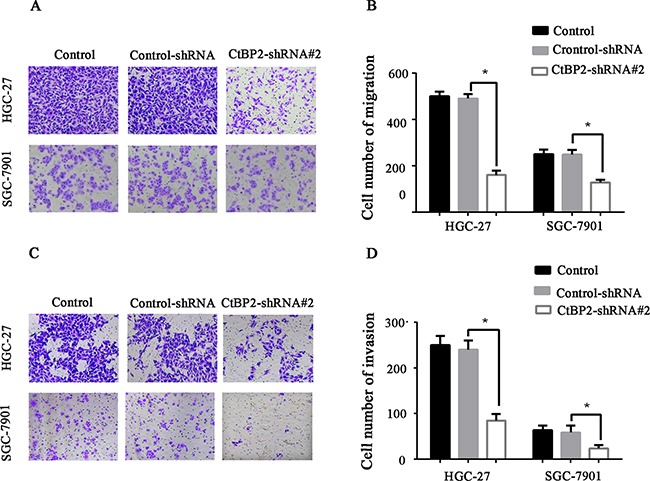
Transwell migration **A, B**. and invasion **C, D**. assays showed that knockdown of CtBP2 expression significantly reduced migration and invasion in both HGC-27 and SGC-7901 GC cells (* p < 0.05).

Cell proliferation, migration, and invasion in the two GC cell lines were evaluated. The HGC-27 and SGC-27 cell lines showed lower proliferation rates after transfection with the CtBP2-shRNA#2, as indicated by colony formation and CCK-8 cell proliferation assays (p < 0.05) (Figure 4A, 4B, 4C, 4D). Flow cytometry cell cycle analysis showed that knockdown of CtBP2 increased the percentage of G1 phase cells and decreased the percentage of S phase cells in GC cells (Figure 4E, 4F). For the migration and invasion assays, fewer CtBP2-shRNA#2-treated GC cells migrated through the chamber, with or without Matrigel, compared with control-shRNA and normal control cells (p < 0.05) (Figure 5).

### Silence of CtBP2 inhibited GC tumorgenesis *in vivo*

**Figure 6:**
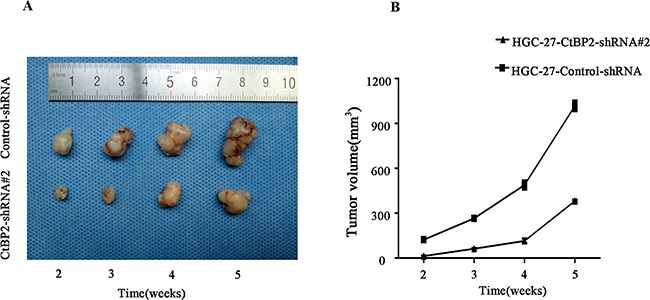
Knockdown of CtBP2 suppresses GC tumourigenesis *in vivo* **A**. Representative pictures of GC tumors excised from BALB/c-nude injected with HGC-27-CtBP2-shRNA#2 (above) and HGC-27-control-shRNA (bellow) after 2,3,4,5 week-tumor-formation respectively. **B**. The growth rate of tumourigenesis *in vivo* after GC cells injection was shown in the growth curve (mean ± SD, p < 0.05).

The role of CtBP2 in GC tumorgenesis of HGC-27 cells was investigated in nude mice model. CtBP2-shRNA#2 and control-shRNA infected HGC-27 cells formed tumors in all BALB/c nude mice. The average tumor volume of the control-shRNA group was significantly larger than that of the CtBP2-shRNA#2 group and the GC growth rate *in vivo* of CtBP2-shRNA#2 group was lower (p < 0.05) (Figure 6).

## DISCUSSION

The role of CtBPs in tumorigenesis was first discovered in studies of the E1A oncogene [19]. CtBP2 is a member of the CtBP family, a group of evolutionarily conserved transcriptional corepressors [13]. Mounting evidence has confirmed that CtBP2 plays an important role in tumorigenesis, including differentiation, cell proliferation, and apoptosis [20, 21]. CtBP2 promotes EMT indirectly [22]. In addition to the repression of E-cadherin, CtBP2 represses PTEN tumor suppressor activity [13, 23]. CtBP2 also acts as an antagonist of apoptosis [22]. CtBP2 was originally investigated in the context of prostate cancer, and recent reports indicate that CtBP2 can modulate androgen receptor activity to promote prostate cancer progression [16]. Indeed, overexpression of CtBP2 has been found in multiple cancers, including ovarian cancer, breast cancer, and hepatocellular carcinoma [17, 18, 24].

In the present study, TMA, IHC, and WB analyses showed that CtBP2 expression was upregulated in GC cell lines and tissues and was closely associated with malignant behaviours and poor prognosis. The levels of CtBP2 expression were significantly correlated with GC differentiation, TNM stage, and vascular and lymphatic invasion in our experiments, partially accounting for the prognostic value of CtBP2 expression in GC patients. These findings are consistent with those of previous studies [13, 16].

The CtBP2 expression in HGC-27 and SGC-7901 GC cell lines was silenced after transfected with CtBP2-shRNA#2. Silence of CtBP2 resulted in downregulation of PCNA, as well as cell cycle arrest, which could account for CtBP2 overexpression increasing GC growth rate. Based on CCK-8, colony formation assays and subcutaneous tumorigenesis in nude mice model, the significant decrease in the cell proliferation rate was consistent with the idea that high levels of CtBP2 expression promote GC tumorigenesis and progression. CtBP2 depletion induced the reversion of EMT and upregulated PTEN, which was consistent with former studies [13, 22, 23]. EMT was characterized as an important program during tumor progression involving in invasive and migratory abilities[24, 25]. PTEN is a critical tumor suppressor involved in many tumor types and loss of PTEN is associated with metastasis and correlated with poor prognosis of GC [26, 27]. Trans-well assay showed silencing of CtBP2 decreased migratory and invasive ability in the GC cell lines. The change might partly explained clinical data analyses that CtBP2 expression was closely associated with TNM stage as well as vascular and lymphatic invasion in GC [17, 28].

In summary, the highly regulated expression of CtBP2 in GC tissues was confirmed to be associated with malignant behaviours and poor prognosis in GC patients. Furthermore, shRNA-mediated silencing of CtBP2 inhibited cell proliferation, migration, and invasion in GC cell lines. This study provided the first analysis of the expression and biological effects of CtBP2 in GC, laying the groundwork for identifying the molecular mechanisms and novel treatments for GC. Further research will be needed to support and explain our findings, and we believe CtBP2 has the potential to become a high-efficacy target.

## MATERIALS AND METHODS

### Patients and tissue samples

This research was approved by the local ethics committee. GC tissues and corresponding adjacent normal tissues were obtained from 352 patients with GC who had undergone surgery at Fudan University Shanghai Cancer Centre, Shanghai, China, between 2010 and 2011. Written informed consent was received from all patients. Tissue specimens were separately formalin fixed and paraffin embedded or snap frozen in liquid nitrogen (N_2_) until use. The average age of this group was 61.41 years (range: 21- 84 years), and the patients consisted of 275 males and 77 females. The clinical data for each patient (i.e., clinicopathological characteristics, including age, gender, differentiation, tumor size, Borrmann type, and TNM stage, are shown in Table 1) and follow-up records were acquired from their medical records using our computerised documentation system (ChiBASE) and telephone investigations. The last follow-up was performed on April 30, 2015. GC stage was classified in accordance with the 7^th^ edition of TNM staging (UICC 2009). Patients with UICC stages III or IV were recommended for either additive or adjuvant chemotherapy.

### Tissue microarray (TMA) analysis and immunohistochemistry (IHC) analysis

Three-hundred fifty-two GC and matched, tumor-adjacent tissues were used for tissue microarray (TMA) analysis. Representative core tissue samples (2.0 mm in diameter) were taken from paraffin-embedded sections and deposited in recipient paraffin blocks individually for TMA construction (Shanghai Outdo Biotech, Shanghai, China).

After cutting into 4-μm sections, the TMA paraffin blocks were deparaffinised and rehydrated using a graded alcohol series. H2O2 (3%) and normal goat serum were used to retrieve and block endogenous peroxidase activity. Next, the sections were incubated with an anti-CtBP2 antibody (dilution: 1:300; ab128871, Abcam, Cambridge, UK) overnight at 4°C, followed by incubation with an HRP-conjugated goat anti-rabbit secondary IgG at 37°C for 10 min. Signal was developed using 3, 3′-diaminobenzidine as the detection substrate (Nichirei) for 5 min and counterstained with 10% Mayer's haematoxylin.

CtBP2 expression was evaluated by two experienced pathologists without knowledge of the corresponding clinical information. Staining intensity was scored using the following criteria: 0 (negative), 1 (weak), 2 (moderate), and 3 (strong). Positively stained cells were quantified as a percentage of the total cells (0-100%). The final score was calculated by summing the intensity scores and determining a percentage as follows: 0 (no staining) – 300 (100% of cells scored strong). The 352 GC cases were classified into two groups based on the final score of CtBP2 expression: CtBP2 expression-low (final score <200, n = 196, 56%) and CtBP2 expression-high (final score > or = 200, n = 156, 44%).

### Cell lines and cell culture

The human GC cell lines HGC-27 and SGC-7901 and the normal gastric cell line GES1 were obtained from the Type Culture Collection cell bank of the Chinese Academy of Sciences Committee (Shanghai, China). We maintained the cell lines in RPMI-1640 medium (HyClone, Logan City, Utah, USA) supplemented with 10% FBS (foetal bovine serum) at 37°C in the presence of 5% CO2 in a humidified incubator.

### Western blotting (WB) analysis

WB analysis was performed as previously described [29] to confirm the expression of CtBP2 and GAPDH in the GC cell lines, GC tissues, and matched adjacent normal tissues. Tissue samples for WB were stored in N_2_. RIPA lysis buffer (Beyotime Institute of Biotechnology, Jiangsu, China) was used to extract total protein from tissue samples and cell lines. Protein concentration was measured using a BCA protein assay kit (Beyotime Institute of Biotechnology). Equal amounts of protein were separated using 10% SDS/polyacrylamide gel electrophoresis (SDS-PAGE) (Beyotime Institute of Biotechnology) and were transferred onto polyvinylidene difluoride (PVDF) membranes (Millipore, Billerica, MA, USA). The membranes were probed with an anti-CtBP2 antibody (1:20,000; Abcam, Cambridge, UK). Expression of CtBP2 was determined using a horseradish peroxidase (HRP)-conjugated goat anti-rabbit IgG antibody (1:1,000; Proteintech, Rosemont, IL, USA). After stripping, the membranes were reprobed with an anti-GAPDH mouse monoclonal antibody (1:1,000; Proteintech) overnight at 4°C as a loading control. The bands were visualised using an ECL system (Thermo Fisher Scientific Inc., Waltham, MA, USA) and quantified by densitometry.

### Cell transfection

The HGC-27 and SGC-7901 cell lines were treated separately as follows. First, the lines were treated with cisplatin (37.5, 150, 600, 2400, or 9600 ng/ml) diluted in RPMI 1640. Next, the cell lines were transfected with control-shRNA or CtBP2-shRNA according to the manufacturer's instructions (Genechem, Shanghai, China). The CtBP2-specific shRNA target sequences were as follows: CtBP2-shRNA#1 – 5′-CTTTGGATTCAGCGTCATA-3′; CtBP2-shRNA#2 – 5′-CTGCAATCTCAACGAACAT-3′; CtBP2-shRNA#3 – 5′-TGAGAGTGATCGTGCGGAT-3′; and CtBP2-shRNA#4 – 5′-GACAGAATTTGTGAAGGTA-3′. Six hours after transfection, the medium was replaced. Cells were collected for WB, CCK-8, colony formation, and Transwell migration and invasion assays after transfection for 48 h. Untreated cells were also prepared as a negative control. For tumor formation assay of GC in mice model, stably silence CtBP2 and control HGC-27 cell lines were constructed by lentiviral vector transduction as previously described [30]. Stable cell lines were respectively selected with 0.5 mg/mL puromycin for 10 days.

### Colony formation assays

Two groups of cells (HGC-27 and SGC-7901; 0.5×10^3^/well) were plated in 96-well cell culture plates (Corning Inc., Corning, NY, USA) and then cultured for 1 to 2 weeks or until colony formation in each group was complete. Next, the cells were fixed with 10% formaldehyde for 10 min and stained with crystal violet staining solution (Beyotime Institute of Biotechnology) for 30 min before colonies were counted.

### CCK-8 cell proliferation and flow cytometry analysis

CCK-8 (Cell Counting Kit-8; Dojindo, Kuma-moto, Japan) was used to measure cell proliferation according to the manufacturer's instructions. Briefly, GC cells (5×10^3^/well) were seeded in 96-well cell culture plates in a volume of 100 μL and then grown overnight. Next, the medium was removed from each well, and 10% CCK-8/culture medium was added for 2 h. The assays were performed in triplicate and repeated three times. To investigate the effects of CtBP2 on GC cell cycle, flow cytometry analysis was performed. The detailed experimental procedure has been previously described [31], and the assays were performed three times.

### Transwell invasion and migration assays

Migration and invasion were measured using trans-well assays with a modified double chamber (24-well cell culture cluster with an 8.0-μm pore size; Costar, Cambridge, NY, USA). The inserts were coated with or without Matrigel (BD Biosciences, Bedford, MA, USA) for the cell invasion and migration assays, respectively. A total of 1×10^5^ cells were added to the top chamber in 150 μL RPMI 1640 without FBS, and the lower chamber contained 1 ml medium containing 10% FBS. After a-24-h incubation, the cells remaining in the top chamber were removed. Crystal violet staining solution (Beyotime Institute of Biotechnology) was used to stain cells that had invaded or migrated. Stained cells were imaged and counted. At least 6 random fields (magnification ×200) in each filter were calculated, and the assays were performed three times.

### GC tumorigenesis assay *in vivo*

A number of 5 × 10^6^ HGC-27 cells (stably silence CtBP2 and control HGC-27 cell lines) were subcutaneously injected into the flanks of 5-week-old male BALB/c nude mice (Animal Center of the Medical College of Nantong University, Nantong, China). All mice were fed and raised under SPF conditions. All *in vivo* experimental protocols were approved by Use Committee for Animal Care and operated according to institutional guidelines. Respectively, After 2, 3, 4 and 5 weeks, the mice were sacrificed and tumors were dissected. The tumor sizes were measured and volume was calculated with following formula: V (volume, mm^3^) = 0.5×L (length, mm) ×W^2^ (width, mm^2^).

### Statistical methods

Statistical analyses were performed using the SPSS version 21.0 statistical software package (SPSS, IBM, USA). Figures were constructed using the GraphPad Prism 5.0 software program (La Jolla, CA). The data are presented as the means ± SD. χ^2^ tests were used to compare the clinicopathological data. The Kaplan-Meier method was used to calculate the survival curves, and the log-rank test was used for univariate analysis of the differences between the clinical factors. The multivariate Cox proportional hazards model was used when meaningful factors (p < 0.05 in univariate analysis) were selected to determine which clinicopathological variables were independently predictive of GC, whereas variables that were highly associated with others were excluded. p < 0.05 was considered statistically significant for all methods.
